# Adolescent on the bridge: Transitioning adolescents living with HIV to an adult clinic, in Ghana, to go or not to go?

**DOI:** 10.1371/journal.pone.0273999

**Published:** 2022-09-29

**Authors:** Ramatu Agambire, Gugu G. Mchunu, Joanne R. Naidoo

**Affiliations:** 1 Discipline of Nursing, School of Nursing and Public Health, College of Health Sciences, University of KwaZulu-Natal, Howard Campus, Durban, South Africa; 2 Department of Nursing, Faculty of Health Sciences, Garden City University College, Kumasi, Ghana; 3 Faculty of Health Sciences, Durban University of Technology, Durban, South Africa; 4 Department of Nursing Science, Faculty of Health Sciences, Nelson Mandela University, Eastern Cape, South Africa; Emory University School of Medicine, UNITED STATES

## Abstract

**Background:**

Children survive into adult life with Human Immunodeficiency Virus (HIV), which previously would have been lethal in early childhood.

**Methods:**

The study aimed to describe the current transitional process for Adolescents Living with HIV (ALHIV) in a resource-limited setting in Ashanti Region, Ghana. The study was an explorative study that used an interpretive paradigm. A semi-structured interview guide was used to interview ALHIV, selected by purposive sampling. The study was conducted at a tertiary hospital in Kumasi. Data were analysed using thematic analysis.

**Results:**

Transitioning of ALHIV was done without any guide; the themes generated were on the process of transition in which they used age (13 and above) and disclosure as the criterion to move ALHIV to the adult clinic. Most adolescents complained about being stigmatised, the attitude of staff, interruption of school and separation anxiety as experiences they went through during the transitioning process. On improving transition, ALHIV felt sexual and reproductive health services, information on treatment, privacy, and support were necessary transition components.

**Conclusion:**

The use of age and disclosure of status as a criterion for transitioning ALHIV affects moving and retaining this age group in HIV management programs in the adult clinics. There is, therefore, an urgent need for a guideline as the current transition process defeats the purpose of providing adolescents with age-specific care in the Adult Clinic.

## Introduction

Globally, failure to offer effective support and acceptable HIV services to adolescents has resulted in a 50 per cent increase in reported AIDS-related deaths within this group compared with the 30 per cent decline seen in the general population from 2005 to 2012 [1–3].

For many illnesses detected in childhood, care and therapy will continue throughout life and require the transfer of young people from a pediatric to an adult setting [4]. However, less is known about the burden of HIV and AIDS among these adolescents [5–7]. The transition of ALHIV from pediatric to adult health for services will continue to be a significant issue [8, 9].

Health and development experts globally recognise adolescence as a unique time of transition during which a person needs access to quality health, education, and other social services [10].

Shroufi et al. noted that the risk of death among people living with HIV and awaiting treatment is high. However, the most significant threat is adolescents’ transition to adult clinics. They explained that adolescents usually do not present to Anti-Retroviral Therapy (ART) service after transitioning and are at an increased risk of death because most adolescents do not stay in the HIV programs meant for adults [2, 11].

A literature review was conducted in Ghana on studies done among ALHIV, and few studies were identified but were not on the transition process [11–14]. The reviewed literature revealed that no study had described the transition of ALHIV from the pediatric to the adult clinic in Ghana. Studies [4, 11, 15–18] has been done in other developed countries such as the United Kingdom, Australia, South Africa on the transition process and guidelines designed to help improve the process [19–25].

The study is part of a more extensive study that intends to explore the current transitional care for ALHIV to develop a practice guideline for ALHIV transitioning to adult service in a resource-limited setting in Ghana. This present study aimed to describe the current transitional process for ALHIV and their experiences with transitioning them from the paediatric to the adult clinic.

## Method

### Study setting

The study was conducted in a teaching hospital in Ghana’s most populated and fast-growing town, Kumasi [26]. Data were collected at the HIV centre, which mostly runs on an Out-Patient basis. The Unit offers ART and comprehensive HIV care. The Unit is responsible for the registration and management of all cases of HIV in adults and adolescents older than ten years.

Reviews for Adolescents are scheduled every three months (Mondays) in the adult clinic. Different clinicians see them at each visit, and they collect medication at an onsite pharmacy. There were no support groups or additional services available in the adult clinic.

### Study design

This paper formed part of an extensive study in which qualitative methods were used to collect data to explore the experiences of ALHIV as they transition from the paediatric clinic to the adult clinic. The interviews were semi-structured and not restricted to specific questions so that the ALHIV could express themselves. Information was gotten by asking follow-up questions and justifying previous answers. The study sought to describe and understand the complex nature of transition in a clear, natural, and contextualised way [27].

### Sampling and sample size

Purposive sampling was used to select ALHIV on their scheduled clinic days. Criteria for selecting ALHIV for participation in this study was ALHIV who were between the ages of 13 to 19 years, enrolled in HIV care and taking ART, aware of their HIV diagnosis, either willing to consent to or with consent from caregiver, and were transitioning or had been transitioned during the study period.

The exclusion criteria of the study comprised of ALHIV who were seriously ill as determined by the doctor and in inpatient care. Also, newly enrolled ALHIV were not included since they lacked experience with HIV management program. Also, respondents, less than 18 without their parents or guardians were excluded.

The sample size for the study was thirteen (13), and this was determined by saturation as successive interviews generated similar responses, and no new themes and sub-themes emerged [27–30].

The sample size also was influenced by other studies on transitioning ALHIV in other parts of the world, where the sample size was about the same [27].

### Instrument for data collection

A multidisciplinary team of researchers experienced in HIV care developed a 7-item semi-structured interview guide (S1 File). The second (G.M- PhD, MSc, BN) and third (J.N- PhD, MSc, BN) authors have vast experience in HIV and adolescent issues. Expert opinions were also sought from clinicians involved in the care of ALHIV who had published papers on ALHIV [12, 14, 30].

The study is part of an extensive study in which an integrative review was conducted to gain insight into other transitional care, which guided the researchers in developing the instrument.

Questions were designed to elicit information about the transition process, barriers and facilitators to transition, and how to improve transition. Demographic data were collected, and open-ended questions were asked to encourage respondents to elaborate on their views on transitions with specifics about the current practices. ALHIV interviews were audiotaped and recorded on paper and transcribed later with their consent.

### Data collection process

ALHIV were enrolled onsite during their routine visit. The first author thoroughly explained the details of the study and obtained consent. Written parental consent was obtained from eligible ALHIV who were between 13 to 17 years old; respondents less than 18 without their parents or guardians were excluded. Written informed consent was also obtained from eligible ALHIV 18 years and above.

We considered transition to adult care as the exposure; therefore, adolescents who remained in paediatric care after their 13th birthday were in the paediatric clinic, and adolescents who transitioned to the adult clinic at any age were in the adult clinic.

After consenting to the study, adolescents were interviewed by the first author RA (MPH, BSN, RGN) who was a PhD student at the time of Data collection. This interview was held in a private room within the clinic. Participants were reminded that the process is entirely voluntary and can stop at any time without any implication. The interview lasted from an hour to an hour and thirty minutes. Data was collected for six months (from March 2020 to August 2020).

### Data analysis

All the interviews were transcribed verbatim and analysed concurrently with data collection, following the principles of thematic analysis [28]. The first author (RA) transcribed each audio-recorded interview verbatim. Analysis was based on the thematic analysis by Braun, V., & Clarke, V. (2006), which entailed identifying, analysing, [31] and reporting patterns with the data collected (S2 File).

A codebook (S1 Checklist) contained the codes list—two coders made up of the first author and an independent coder. The independent coder had no prior experience or knowledge of the topic of concern.

### Trustworthiness

Member checks with respondents were done to ensure the study’s trustworthiness [28] while collecting the data. Feedback and confirmation were sought from participants whilst analysing the data. The research team also discussed any discrepancies in the coding and resolved them through consensus, and thus the final set of codes for analysis is based on multiple researchers’ input.

Replication of the study and potential applicability in a similar setting was achieved by a detailed description of the study setting, design, methodology, and the respondents’ background. Further, discussing study findings among authors ensured the auditability of the study [27].

### Consent to participate in study

Information about the research was explained to the adolescent and their parents. Parents and adolescents who agreed to participate in the study gave their consent in writing. Written consent was obtained from adolescents 18 years and parents who accompanied ALHIV less than 18 years to the clinic, and this was done individually with each participant. With their consent, the interviews were audiotaped and recorded on a sheet of paper.

### Ethical approval

Ethical approval was sought and granted by the Committee on Human Research, Publication and Ethics, Kwame Nkrumah University of Science and Technology and KATH, Kumasi (Ref: CHRPE/AP/071/19) and Biomedical Research Ethics Committee, University of KwaZulu-Natal, South Africa (Ref: BE549/18). Institutional permission was also sought and granted by Hospitals. Participants were informed and further briefed on their rights to voluntary participation and withdrawal from the study without consequences. Participants gave consent in writing to both the interview and the audio recording of the interview before data was collected. Participants’ confidentiality was ensured by interviewing them in an enclosed office with no interruption. Information that could reveal the participants’ identities was excluded from the transcripts to ensure anonymity. Participants’ personal identifying information was omitted from the transcripts and replaced with pseudo-names to ensure the anonymity of participants.

## Results

### Participant’s demographic characteristics

Thirteen (13) ALHIV participated in the study, with the mean age being 17. The youngest ALHIV who had transitioned was 13 years, with the oldest ALHIV being 25 years of age. There was a similar sex composition of adolescence in the clinic, thus an equal number of females to males. Two had primary education, three junior high and more than half (7) had senior high or tertiary education, and one had no formal education. Almost all the adolescents were unemployed. Christianity was the major religion of the ALHIV. None of the adolescents was married though a few were in relationships. The detailed demographic characteristics of the participants are shown in the S1 Table.

### Themes & subthemes

ALHIV described their experiences with transitioning from the paediatric to the adult clinic. The themes that emerged were; the process of transition, experiences of ALHIV with the process of transitioning, and improving transition for ALHIV. The three main themes that emerged had sub-themes, as shown in S2 Table.

#### Theme 1: Process of transition

The process of transitioning ALHIV is unstructured. The process starts when each ALHIV is 13 years old and informed of the transition, this is followed by disclosing their status and then the physical movement to the adult clinic (S1 Fig). **The** S1 Fig **describes the transition processes from the paediatric clinic to the adult clinic**.

*Subtheme 1*: *Adolescent age*. There were no criteria for determining readiness for transitioning ALHIV. The primary determinant was the age of the adolescents (13 years and above). According to ALHIV, after their 13th birthday, they were told to join the adult clinic.

“The process started with the doctor telling me about the maturity age (13), so I would be moved from the children to the older people; then, I was made to invite my parents when they told me I had HIV”.

***(Mabel)***

“When you reach adolescence, usually 13 and above, they talk to you about the diagnosis (HIV), and then they will tell you that you will be moved to the adult clinic because you are mature now”.

***(Cindy)***

*Subtheme 2*: *Disclosure*. The Healthcare workers disclose the status of adolescents discretely, either alone or in the presence of their guardians or parents. The parents or guardians of the ALHIV are invited to be part of the disclosure process. However, the ALHIV felt the timing was late and should not be before leaving the paediatric clinic. Several ALHIV were not ready to share the information with anyone.

Some of the responses were:

“I was not aware I had HIV until I was moved to the adult clinic. My mother told me I had it from birth, but why are they telling me now? I have not told anyone about this illness. My boyfriend is not aware, or else he will leave me”.

***(Russian)***

“It was very tough and painful when they told me I had HIV. At my age, I have such illness. However, with encouragement from my mother and the nurses, I could cope with it and console others with the disease”.

***(Serwaa)***

*Subtheme 3*: *Moving to the adult clinic*. Most of the adolescents refused to move to the adult clinic after disclosure. The few who moved complained that they did not feel comfortable in their new environment. The children’s clinic allowed them to express themselves, but the adult clinic is strict, making it difficult to speak.

“I have not been able to move, but I will soon. The nurses keep sacking me to go to the adult clinic. If I move to the adult clinic and the nurses there do not treat me well, I will not return to the clinic again; I will die soon”.

***(Akoma)***

“They formed groups, of which they told me to join and they spoke to us about the transfer during a group meeting. They gave us handouts and made us fill out forms, and then the doctor told us that they would be moving us soon to the adult clinic. I left for the adult clinic a few months afterwards”.

***(Amina)***

#### Theme 2: Experiences of ALHIV with the process of transitioning

The ALHIV shared their experiences with the process of transition. Some had moved to the adult and come back to the paediatric clinic after these experiences. Some were still contemplating either to go or not to go.

*Subtheme 1*: *Perception of stigma*. According to the Adolescents, they suffer Stigmatisation both at the adult clinic and sometimes when they have to access services outside the HIV clinic, and their status is known.

“The older patients look at me differently at the adult clinic because I am young. Sometimes, I hear them contemplating how children/adolescents got infected. They are many, and I always look over my shoulders, so one recognizes me”.

***(Amina)***

“When I access other services, and they get to know I have HIV, then everything changes. At the laboratory, they do not usually wear gloves when taking samples of other people, but once it is my turn, they will wear gloves”.

***(Serwaa)***

*Subtheme 2*: *Staff attitude*. According to the adolescents, the attitude of the health workers at the adult clinic is terrible as they do not give them the needed care and attention at the adult clinic. Some gave accounts of the treatment meted out to them.

“Sometimes when I ask the nurses questions, they do not respond. Other times they get angry. They keep changing the nurses, and I meet different people. I have to keep repeating myself, and it is as if they do not care”.

***(Akua)***

“Some of the staff are not nice, approaching them for help is difficult. I stopped coming to the clinic for months, but I had to start again when I fell sick”.

***(Cindy)***

*Subtheme 3*: *Interruption of work/school schedule*. For most adolescents in school, the monthly review schedule makes them miss school to keep the appointment. Sometimes they have to visit the clinic more than once to finish the processes and get their medications, while others miss work to get their medications.

“It is challenging. I have to miss classes on Mondays, and when I continuously skip classes, my friends and teachers think I am lazy”.

***(Dela)***

“Some of us are workers while others are students. Sometimes I have to leave work to come to the clinic. I left my job and came here early to go back without anyone noticing my absence, but I spent the whole day here. When I get back, my coworkers will ask where I am coming from, and I have to lie”.

***(Mabel)***

*Subtheme 4*: *Separation anxiety*. It becomes challenging for adolescents to break off their relationship and move to the adult clinic as they indicated that, they have been with the staffs at the paediatric setting from childhood through adolescence in happy and sad moments, and they understand them better.

“I have not gone to the adult clinic because of the doctor taking care of me in the paediatric clinic, he is adorable, but I do not know the new doctor. That is why I have refused to move to the adult clinic”.

***(Akoma)***

“I have known the staff since childhood. I share my problems with them, and the doctor is like a father. How would I relate to the new people in the adult clinic?”

***(Amina)***

#### Theme 3: Improving Transition for ALHIV

The ALHIV suggested a few strategies for improving the transition process. They attributed the transition process to the inconsistency in accessing healthcare and non-adherence to treatment.

*Subtheme 1*: *Information on treatment and medication*. The adolescents emphasised the need for information about the drugs and their treatment. For most of them, there are instances in which they take the medicines not because they know why but out of fear of dying.

“I need more information about the drugs. It will help me deal with the side effects. I take my pills before or after meals; it depends on how I feel. Although I take the pills, I fear I will die, and it is demoralizing”.

***(Cindy)***

“The multiple pills have been reduced at the adult clinic, and that is all I know. It is easier to take the pills now, but I mostly forget to take them for days”.

***(Mabel)***

*Subtheme 2*: *Privacy and confidentiality*. There was a need for privacy during the consultation, irrespective of the patient’s age. This will help them to express themselves without any reservations.

“I cannot talk about my sexuality in the presence of others; I am shy. The consulting rooms always have other health workers seated doing different things. There is no privacy within the clinic, and it is frustrating”.

***(Cindy)***

“Some of us have not disclosed our status to others, and if I come to the clinic with someone who does not know through the nurse’s indiscretion, she will reveal my HIV status to that person, which can be embarrassing”.

***(Russia)***

*Subtheme 3*: *Provision of sexual and reproductive health service*. As ALHIV matures into adulthood, the urge to get into relationships and have sex increases.

“I need information on my sexuality, STIs, pregnancy, etc. You know, not everyone can abstain, and I cannot, meanwhile, they educate us mainly at the OPD, but I am shy to ask if I have questions”.

***(Emelia)***

“The nurses should talk to us about our sexuality to help us make the right choices. I have a boyfriend; we were together before being told I had HIV. We have sex. I am afraid to ask about sex since they think I am a bad girl”.

***(Serwaa)***

*Subtheme 4*: *Support systems*. ALHIV requires support. The only source of support is from their parents or guardians, who are mostly either sick, dead, or poor. They have difficulty supporting themselves emotionally, psychologically, financially, and physically.

“Usually, the drugs are not expensive, but the test and other costs make them expensive. I have to do laboratory investigations, which is costly, and without money, I cannot do it. I default because I do not have money for some of these expenditures”.

***(Pabi)***

“I need help. I am into vocational training, and if I can get help to complete the training, I can make money and take care of myself. I cannot keep the review dates due to financial difficulties when I do not have money”.

***(Awuni)***

## Discussion

The study explored the process of transitioning ALHIV from the paediatric clinic to the adult clinic and experiences of ALHIV with the process. Transitioning of ALHIV ideally entails a gradual process where adolescents move from the paediatric clinic to the adult clinic. Adolescents must know and accept that health care will shift from the paediatric/adolescent clinic to the adult clinic [19].

While many clinical programs strive to enhance the acquisition of transition-related skills of the adolescents they serve, these programs do not include an assessment of the youths’ transition readiness as part of routine healthcare [20]. To fill this gap in the literature, Zanoni BC et al. 2021 has designed and validated HIV Adolescent Readiness to Transition Scale (HARTS) to help determine when an adolescent is ready to transition to adult care and to identify which adolescents may need further interventions before the transition to improve viral suppression rates after the transition. There is a need for ALHIV to be evaluated for transition readiness using such available tools [21].

The findings of the current study showed that transitioning of ALHIV was unstructured. The process entailed putting the adolescents into groups when they were 13 years old, disclosing their status, and then informing them of the need to move to the adult clinic. Most Adolescents in this group did not know their status and why they were taking the medication. Their status is disclosed, and they follow up with the need to move to the adult clinic; this completes the transition process. Unfortunately, because there are no guidelines on the transition processes, it differs depending on who (health care worker) is taking them. This reiterates the issues raised by Kallem S et al., 2011; Ankrah D et al., 2016; Kenu E et al., 2014 and Anthony E. et al., 2015 on the need for guidelines as the absence of guidelines and trained personnel on transitioning ALHHIV in Ghana affects the process [11–14]. In their study, Gilliam P et al., 2011 highlighted the need for transition to be purposeful and planned to help improve access to healthcare for ALHIV [4].

In other countries like South Africa, the issue is different. There are policies for HIV/AIDS treatment and care services and treatment guidelines to help implement programs [32, 33]. However, in Ghana, these policies do not exist. Once an adolescent is 13 years or more, they are prepared to join the adult clinic without any pre-assessment and leave the ALHIV lingering between the paediatric and adult clinic [34, 35].

A U.S.-based study on paediatric and adult providers suggested that planning transitioning for ALHIV will help improve outcomes [22]. Their process began at least a year earlier and included a meeting between ALHIV and the adult provider, the adult clinic, a tour. After transitioning, conversations about differences between the paediatric and adult clinics had better outcomes [17, 22].

Individual differences in determining maturity pose a significant challenge in deciding how these adolescents are transferred. There is difficulty in moving the ALHIV to the adult clinic after transitioning from paediatric to adult health services, which is an essential issue [3]. For many illnesses detected in childhood, care and therapy will continue throughout life, and young people will need to be transferred from the paediatric to an adult setting [1, 3, 8, 9].

The importance of how well these children are transitioned cannot be overrated, especially now that the children with HIV are surviving into adult life, which previously would have been lethal in early childhood [3]. Failure to ensure the satisfactory transition from paediatric to adult services is associated with poor treatment adherence by young people with chronic diseases and disabilities and subsequent poorer outcomes and increased service costs [2, 6].

This study found that most ALHIV refused to come for reviews because their teachers complained about missing class. Also, most of their schoolwork was done on Mondays. The current study findings are similar to a study in Zimbabwe by Mburu, G et al., 2014 in which ALHIV dropped out of treatment because programs at the structural level do not consider the needs of adolescents; they had poor flexibility of clinic opening hours, staff shortage, and lacked a practice guideline [36]. The finding also confirms the work of Ojikutu B et al., 2014, conducted in Nigeria, which concluded that providing high-quality care for adolescents living with HIV is vital to improve outcomes, including reducing loss to follow-up, adherence, and decreasing mortality [37]. There was also fear among the adolescents that their colleagues in school would get suspicious of the missing classes, which, in turn, interrupted health care delivery. In confirmation with Anthony et al., 2015; Chandler et al., 2013; Bleich s et al., 2009, the programs for adolescents should have guidelines that help integrate schools into the treatment programs after the transition [12, 16, 38]. Schools must have policies that allow ALHIV to miss class to access health services. Since health and development experts globally identify access to quality health, education, and other social services as unique needs of adolescents, especially in transition [10].

For most ALHIV, the study revealed inadequate treatment information as a barrier, mainly affecting medication adherence after transitioning to the adult clinic. The young adults are considered matured and allowed to take charge of their illness with less supervision from health providers and guardians. Some ALHIV, however, forgot to take their drugs, especially those in schools, either because they did not want to create any suspicion or raise queries from their peers. Idele and colleagues [6] noted that as adolescents leave home for higher education, they need to transition from dependence on their guardians to independence. Most do not adhere to ART and miss follow-up appointments after leaving home. According to Machado et al. [24], peer educators have noted that Adolescents are angry at themselves and their parents on how they were infected, which was consistent with the current study. ALHIV do not take their medications because they feel they are doing well or think they are punishing their parents who gave them the disease, especially adolescents infected perinatally [25].

Another underlying factor causing delays and resistance in transition was the unwillingness of the adolescents to break ties with their adolescent health care providers. During their stay at the paediatric clinic, the adolescents and their healthcare providers develop a bond of trust, making it easy to confide in them. Leaving the adolescent health care providers is seen by some as losing a family, making transitioning difficult. This confirms some studies within Sub-Saharan Africa where the transition to an adult care setting is a challenge for most ALHIV because of the fear of losing the stable and long-term relationships built with their pediatric or adolescent healthcare team [6, 13, 18, 19, 22]. These often lead to non-adherence and default in clinical visitations.

According to Katusiime, C. et al. 2013 and Miles, K. et al. 2004, clinicians must strive to have a nonjudgmental approach to patients during communication, especially when discussing the sexual behaviours of ALHIV [19, 23]. When spoken to judgmental, adolescents often disengage from care, and their needs differ. As these adolescents grow and experience changes in their bodies and emotions, they need counselling to understand what is happening to them and how to take care of themselves properly. Most adolescents get into relationships without the needed guidance to cope effectively with the disease and their newfound lives.

During the transition, there is little or no support for the adolescents, making the transition process cumbersome. They require support as they go through the transition process to help them cope with the changes they will experience. This should entail follow-ups and psychological support to understand what is happening. During the transition to adult clinics, caring tasks can include verbal and body language that reassures the patient of the necessary support should the need arise [20, 24]. Financial assistance (an ALHIV fund) was essential to help support them financially. Many adolescents complained about not getting money for their transportation, medications, feeding, and the various laboratory tests they do before treatment. These accounted for the many months of non-adherence and the high default rate. A transition readiness assessment is essential and must test available tools among ALHIV to help determine the readiness of adolescents for this crucial step of their growth trajectory [21].

However, this study had some limitations. The use of small sample size from just a single facility might affect the study’s generalisation. Some interviews were conducted in Twi, the vernacular for the area; it is necessary to concede further that the translation process might have distorted some of the data and, accordingly, information may have inadvertently and unavoidably been misrepresented or lost in translation.

## Conclusion

The study was conducted based on the need for data on the current transition process for ALHIV as the lack of published research on the subject of transition of ALHIV in Ghana though a limitation as comparison in context (Ghana) was absent it also served as a motivation for the current study.

The process of transitioning ALHIV from the paediatric to the adult clinic was unstructured. The use of age and disclosure of status as a criterion for transitioning ALHIV had an implication for moving and retaining this age group in HIV management programs in the adult clinics. The transition process calls for collaboration between ALHIV, the family and health care workers to plan a reasonable timeline to start and complete the transition process. Successful transitioning requires that adolescent care teams and stakeholders develop a guideline based on the needs of the adolescents during the transition process. There is an urgent need for a guideline as the current transition process does not provide adolescents with age-specific care in the Adult Clinic. It is required that further studies are done to determine factors that contribute to transition readiness for adolescents living with HIV and how an adolescent-friendly service can contribute to the transition process.

## Supporting information

S1 Table Demographic characteristics of participants. (DOCX)

S2 Table Themes and sub-themes. (DOCX)

S1 Fig Process of transitioning ALHIV. (DOCX)

S1 File Semi-structured interview guide. (PDF)

S2 File Code book. (PDF)

S1 Checklist COREG guideline. (DOCX)

## References

[1] WHO, 2013. Health For The World’s Adolescents A Second Chance In The Second Decade. 10.1016/j.jadohealth.2014.10.260 25530601

[2] Shroufi A., Ndebele W., Nyathi M., Gunguwo H., Dixon M., Saint-Sauveur J. F., et al. 2015. Risk Of Death Among Those Awaiting Treatment For Hiv Infection In Zimbabwe: Adolescents Are At Particular Risk. *J Int Aids Soc*, 18, 19247. doi: 10.7448/IAS.18.1.19247 25712590 PMC4339242

[3] Stricker S. M., Fox K. A., Baggaley R., Negussie E., De Pee S., Grede N. et al. 2014. Retention In Care And Adherence To Art Are Critical Elements Of Hiv Care Interventions. *Aids Behav*, 18 Suppl 5, S465–75. doi: 10.1007/s10461-013-0598-6 24292251

[4] Gilliam P. P., Ellen J. M., Leonard L., Kinsman S., Jevitt C. M. & Straub D. M. 2011. Transition Of Adolescents With Hiv To Adult Care: Characteristics And Current Practices Of The Adolescent Trials Network For Hiv/Aids Interventions. *J Assoc Nurses Aids Care*, 22, 283–94. doi: 10.1016/j.jana.2010.04.003 20541443 PMC3315706

[5] WHO, 2016. <Global-Aids-Update-2016_En_2.Pdf>.

[6] Idele P., Gillespie A., Porth T., Suzuki C., Mahy M., Kasedde S. et al. 2014. Epidemiology Of HIV And AIDs Among Adolescents: Current Status, Inequities, And Data Gaps. *Jadis Journal Of Acquired Immune Deficiency Syndromes*, 66, S144–S153. doi: 10.1097/QAI.0000000000000176 24918590

[7] Orban L. A., Stein R., Koenig L. J., Conner L. C., Rexhouse E. L., Lewis J. V. et al. 2010. Coping Strategies Of Adolescents Living With Hiv: Disease-Specific Stressors And Responses. *Aids Care*, 22, 420–30. doi: 10.1080/09540120903193724 20146110

[8] Bailey H., Cruz M. L. S., Songtaweesin W. N. & Puthanakit T. 2017. Adolescents With Hiv And Transition To Adult Care In The Caribbean, Central America And South America, Eastern Europe And Asia And Pacific Regions. *J Int Aids Soc*, 20, 21475. doi: 10.7448/IAS.20.4.21475 28530040 PMC5577698

[9] Badejo O. A., Menson W. N. A., Sam-Agudu N. A., Pharr J., Erekaha S., Bruno T., et al. 2018. Pediatric To Adult Healthcare Transitioning For Adolescents Living With Hiv In Nigeria: A National Survey. *Plus One*, 13, E0198802. doi: 10.1371/journal.pone.0198802 29894519 PMC5997346

[10] UNICEF & U. N. C. S. F. 2013. Towards An Aids-Free Generation—Children And Aids. *NY*: *Unicef*; Sixth Stocktaking Report,

[11] Ankrah D. N. A., Koster E. S., Mantel-Teeuwisse A. K., Arhinful D. K., Agyepong I. A. & Lartey M. (2016). Facilitators And Barriers To Antiretroviral Therapy Adherence Among Adolescents In Ghana. *Patient Prefer Adherence*, 10, 329–37. doi: 10.2147/PPA.S96691 27042024 PMC4801129

[12] Anthony E., Nugent N., Amoah C., Norman B., Antwi S., Ocran J., et al. 2015. Quality Of Life Among Ghanaian Adolescents Living With Perinatally Acquired Hiv: A Mixed Methods Study. *Aids Care*, 28, 460–4. doi: 10.1080/09540121.2015.1114997 26643735 PMC4764441

[13] Kallem S., Renner L., Ghebremichael M. & Paintsil E. 2011. Prevalence And Pattern Of Disclosure Of HIV Status In HIV-Infected Children In Ghana. *Aids And Behavior*, 15, 1121–1127. doi: 10.1007/s10461-010-9741-9 20607381 PMC2989337

[14] Kenu E., Obo-Akwa A., Nuamah G. B., Brefo A., Sam M. & Lartey M. 2014. Knowledge And Disclosure Of Hiv Status Among Adolescents And Young Adults Attending An Adolescent Hiv Clinic In Accra, Ghana. *Bmc Research Notes*, 7, 1–6. 25424862 10.1186/1756-0500-7-844 PMC4256736

[15] Boockvar K. & Vladeck B. C. 2004. Improving The Quality Of Transitional Care For Persons With Complex Care Needs. J Am Geriatr Soc, 52, 855–6; Author Reply 856 doi: 10.1111/j.1532-5415.2004.52230_15.x 15086688

[16] Chandler, C. L. & Ngoksin, A. E. 2013. *Lost In Transitions*: *Current Issues Faced By Adolescents Living With Hiv In Asia Pacific*, The Asia Pacific Network Of People Living With Hiv (Apn+).

[17] Coleman E. A. & Boult C. 2003. Improving The Quality Of Transitional Care For Persons With Complex Care Needs. *J Am Geriatr Soc*, 51, 556–7. doi: 10.1046/j.1532-5415.2003.51186.x 12657079

[18] Désiré Lucien Dahourou Chloé Gautier-Lafaye, Teasdale Chloe A., Renner Lorna, Yotebieng Marcel, Desmonde Sophie, et al. 2017. Transition From Paediatric To Adult Care Of Adolescents Living With Hiv In Sub-Saharan Africa: Challenges, Youth-Friendly Models, And Outcomes. *Journal Of The International Aids Society* 2017, 20(Suppl 3):21528. doi: 10.7448/IAS.20.4.21528 28530039 PMC5577723

[19] Katusiime, C., Parkes-Ratanshi, R. & Kambugu, A. 2013. *Transitioning Behaviourally Infected Hivpositive Young People Into Adult Care*: *Experiences From The Young Person’s Point Of View*.

[20] Scal P., Evans T., Blozis S., Okinow N., & Blum R (1999). Trends in transition from pediatric to adult health care services for young adults with chronic conditions. Journal of Adolescent Health, 24(4), 259–264. doi: 10.1016/s1054-139x(98)00127-x 10227345

[21] Zanoni BC, Archary M, Sibaya T, Musinguzi N, Kelley ME, McManus S, et al. Development and validation of the HIV adolescent readiness for transition scale (HARTS) in South Africa. J Int AIDS Soc. 2021 Jul;24(7):e25767. doi: 10.1002/jia2.25767 . 34235876 PMC8264413

[22] Kirby E., Broom A. & Good P. 2014. The Role And Significance Of Nurses In Managing Transitions To Palliative Care: A Qualitative Study. *BMJ Open*, 4. doi: 10.1136/bmjopen-2014-006026 25270859 PMC4179576

[23] Miles K., Edwards S. & Clapson M. 2004. Transition From Paediatric To Adult Services: Experiences Of Hiv-Positive Adolescents. *Aids Care*, 16, 305–14. doi: 10.1080/09540120410001665312 15203424

[24] Machado D. M., Galano E., De Menezes Succi R. C., Vieira C. M. & Turato E. R. 2016a. Adolescents Growing With Hiv/Aids: Experiences Of The Transition From Pediatrics To Adult Care. *Braz J Infect Dis*, 20, 229–34. doi: 10.1016/j.bjid.2015.12.009 26945104 PMC9425401

[25] Skilbeck J. & Payne S. 2003. Emotional Support And The Role Of Clinical Nurse Specialists In Palliative Care. *Journal Of Advanced Nursing*, 43, 521–530. doi: 10.1046/j.1365-2648.2003.02749.x 12919270

[26] Ghana Statistical Services Gss 2010. Demographic And Health Survey. *Report*.

[27] Nk Denzin, Ys Lincoln. 2011, The Sage Handbook Of Qualitative Research: Sage.

[28] Df Polit, Ct Beck. 2008, Nursing Research: Generating And Assessing Evidence For Nursing Practice: Lippincott Williams & Wilkins;

[29] Rk Yin. (2017) Case Study Research And Applications: Design And Methods: Sage Publications.

[30] Eg Guba, Ys Lincoln. (1989) Fourth Generation Evaluation: Sage; 1989.

[31] Braun V., & Clarke V. (2006). Using Thematic Analysis In Psychology. *Qualitative Research In Psychology*, 3(2), 77–101.

[32] National AIDS/STI Control Programme (2013).

[33] Bygrave H., Mtangirwa J, Ncube K, Ford N, Kranzer K, Munyaradzi D. 2012. Antiretroviral Therapy Outcomes Among Adolescents And Youth In Rural Zimbabwe. Ed. *Plos One*. 2012;7(12):E52856. doi: 10.1371/journal.pone.0052856 23285204 PMC3527625

[34] Ghana AIDS Commission 2013. National HIV and AIDS, STI Policy.

[35] National Department Of Health 2010, Clinical Guidelines for the Management of HIV &AIDS in Adults and Adolescents.

[36] Mburu G., Ram M., Oxenham D., Haamujompa C., Iorpenda K. & Ferguson L. 2014. Responding to adolescents living with HIV in Zambia: A social-ecological approach. *Children and Youth Services Review*, 45, 9–17.

[37] Ojikutu B., Higgins-Biddle M., Greeson D., Phelps B. R., Amzel A., Okechukwu E., et al. 2014. The association between quality of HIV care, loss to follow-up and mortality in pediatric and adolescent patients receiving antiretroviral therapy in Nigeria. *PLoS One*, 9, e100039. doi: 10.1371/journal.pone.0100039 25075742 PMC4116117

[38] Bleich S. N., Murray C. J. L. & Özaltin E. 2009. How Does Satisfaction With The Health-Care System Relate To Patient Experience? *Who Bulletin Of The World Health Organization*, 87, 271–278. doi: 10.2471/blt.07.050401 19551235 PMC2672587

