# Supplementary material for: Adolescent on the bridge: Transitioning adolescents living with HIV to an adult clinic, in Ghana, to go or not to go?
Source: PLoS One. 2022 Sep 29;17(9):e0273999. doi: 10.1371/journal.pone.0273999 (PMC9522288; doi:10.1371/journal.pone.0273999)
Supplement: S1 Table — (DOCX) [file pone.0273999.s001.docx]

**S1 Table: Participant's Demographics**

| **Respondent No** | **Pseudo names** | **Age range**  **(years)** | **Educational background** | **Occupation** | **Gender** | **Age of Disclosure** | **Number of months after disclosure** |
| --- | --- | --- | --- | --- | --- | --- | --- |
| 1 | Esi | 13-15 | Elementary | Unemployed | Female | 14 | 5 |
| 2 | Akoma | 16-18 | Junior High | Trader | Male | 16 | 6 |
| 3 | Serwaa | 13-15 | Elementary | Unemployed | Female | 13 | 36 |
| 4 | Akua | >18 | Illiterate | Employed | Female | 18 | 36 |
| 5 | Emefa | 13-15 | Junior High | Trader | Male | 15 | 4 |
| 6 | Dela | ≥18 | Tertiary | Unemployed | Male | 18 | 10 |
| 7 | Russia | 16-18 | Senior High | Unemployed | Male | 17 | 19 |
| 8 | Cindy | 16-18 | Senior High | Unemployed | Female | 16 | 3 |
| 9 | Amina | >18 | Tertiary | Unemployed | Female | 15 | 36 |
| 10 | Awuni | 16-18 | Senior High | Unemployed | Male | 16 | 24 |
| 11 | Abena | 16-18 | Senior High | Unemployed | Female | 18 | 2 |
| 12 | Pabi | 16-18 | Senior High | Unemployed | Male | 17 | 12 |
| 13 | Mabel | 16-18 | Junior High | Unemployed | Female | 18 | 19 |
