# Supplementary material for: Adolescent on the bridge: Transitioning adolescents living with HIV to an adult clinic, in Ghana, to go or not to go?
Source: PLoS One. 2022 Sep 29;17(9):e0273999. doi: 10.1371/journal.pone.0273999 (PMC9522288; doi:10.1371/journal.pone.0273999)
Supplement: S2 Table — (DOCX) [file pone.0273999.s002.docx]

**S2 Table: Themes and Sub-themes**

| **Themes** | **Subthemes** |
| --- | --- |
| Process of transition | - Adolescent Age - Disclosure - Moving to the adult clinic |
| Experiences of ALHIV with the Process of Transitioning | - Stigma - Staff attitude - Interruption of work/School - Separation anxiety |
| Improving Transition for ALHIV | - Sexual and reproductive health service - Information on treatment and medication - Privacy and confidentiality - Support |
