# Supplementary material for: Adolescent on the bridge: Transitioning adolescents living with HIV to an adult clinic, in Ghana, to go or not to go?
Source: PLoS One. 2022 Sep 29;17(9):e0273999. doi: 10.1371/journal.pone.0273999 (PMC9522288; doi:10.1371/journal.pone.0273999)
Supplement: S1 Fig — (DOCX) [file pone.0273999.s003.docx]

**S3 Fig. above is a Flow chart describing the process of Transitioning Adolescents Living with HIV from the Paediatric Clinic to the Adult Clinic.**

**HCW-Health Care Workers**
