# Supplementary material for: Adolescent on the bridge: Transitioning adolescents living with HIV to an adult clinic, in Ghana, to go or not to go?
Source: PLoS One. 2022 Sep 29;17(9):e0273999. doi: 10.1371/journal.pone.0273999 (PMC9522288; doi:10.1371/journal.pone.0273999)
Supplement: S1 File — (PDF) [file pone.0273999.s004.pdf]

In-Depth Interview

Adolescents Living With HIV

Getting Started

- Welcome the participant to the interview.
- Focus on ensuring the participant feels safe and comfortable.
- Review the participant's consent and assent forms, asking the participant to confirm the signatures on the forms.
- Remind the participant that this process is completely voluntary and if he/she wishes to stop at any time, then he/she may do so without any implications on his/her future care.
- Remind the participant that you are going to have an interview which will focus on transitioning. Ask the participant if he/she has any questions before you get started.
- Now ask the participant questions. If necessary, use probes, however these probes should be limited where possible to minimize demand characteristics.

1. Demographic Characteristics

Age ____________Sex ________ Education ____________

Occupation _____________ Marital status ____________ Religion____________

Age of Disclosure_____________________ Period after Disclosure___________

Years since knowing own HIV status _________ Ever stopped taking ART-___________

1. How will you describe the process of transition?

2. Please tell us what you think about transitioning/transferring you from the paediatric to the adult clinic.
    - Experiences in terms of preparation for transition/initial reaction to transition
    - During the transition process
    - Beyond transition in the adult clinic

3. How has your experience been like during the transition?
4. What factors do you think can influence transition?

5. Please describe any barriers/challenges during your transition (Services, Clinic and Individual)
   - Please tell me more about these challenges; how and why do they impact transition.
   - How are you treated in the health facilities during the transition period?
6. Describe the type of support you will require for a successful transition? What are your needs as ALHIV for a successful transition process? Probe on these areas; Linkage to Care, Sexual and reproductive health, STI &Pregnancy, Default In treatment, ARV Receipt/ Non -Adherence, Access, Utilization

7. Finally, we want to learn from you if you have any comments, concerns or suggestions to improve transition for adolescents living with HIV/AIDS into adult clinic.
   - Please tell us what you want in the health care facilities for betterment of transition services. Probe
   - What are your concerns (if any) about transition of adolescents with HIV?
   - What should health facilities and health care providers do to improve transition process?
   - How do you want to see health facilities operate to encourage adolescents living with HIV to utilize service?

Is there anything else you would like to add on your views about transitioning adolescents living with HIV into adulthood clinics?

Concluding remarks

Thank you so much for sharing your experiences with me. If you have any further queries do feel free to get in touch with me through the number I have provided.
