# Supplementary material for: Adolescent on the bridge: Transitioning adolescents living with HIV to an adult clinic, in Ghana, to go or not to go?
Source: PLoS One. 2022 Sep 29;17(9):e0273999. doi: 10.1371/journal.pone.0273999 (PMC9522288; doi:10.1371/journal.pone.0273999)
Supplement: S2 File — (PDF) [file pone.0273999.s005.pdf]

CODE BOOK FOR ADOLESCENTS LIVING WITH HIV

GLOBAL THEME	BASIC CODES	DESCRIPTION	SAMPLE RESPONSE: R
Transition Process	**Process**	Transitioning readiness is basically determined by age and the willingness of the patient to be transferred	**Respondent 13 said:**<br>*"Before I was taken to the adult clinic, the doctor called me and told me that I had reached my maturity age, so he has to move me from the children to the older people, he didn't add anything."*<br>**Respondent 8 also added:**<br>*"They had some group discussions with those of us they wanted to transfer. We were made to form some groups and they interviewed us as we are doing now. They gave us hand outs and also made us fill some forms before he came to tell us that he is taking us from the children to the adult clinic."*<br>**Respondent 7shared:**<br>*" Ok, first of all, when you reach the adolescent age, they talk to you before, for you to get the understanding, then they tell you that the next time you come , you will go to this place (adult clinic), that's how they do it"*
	**Preparations for Transition**	The ALHIV do not undergo any formal preparation prior to transitioning to the adult clini	**Respondent 8 shared:**<br>*"Ok, what I'm aware of is that, Dr. xxxxx made the selection that when the children are mixed with the older ones, the children suffer and the doctors also do not get much time for the children, so he decided to take care of the children and the older ones will move to the other side. "*<br>**Respondent 12 also added:**<br>*"Before the transition began? I have not been engaged in any process."*

			**R8, R5 said:**<br>*"The doctor told us that when we are 10 to 18 years we will be moved to a different clinic. then they said I should come on a specific date ……"*<br>**R3 and R4 also added this**<br>*"They sit us down and advise us and then add that we have been transferred to the adult clinic"*
	**Reaction to Transition**	Unhappy and unwilling to move, others were willing ates	**Respondent 10 stated**:<br>***"****They want to transfer me to the adult class but am not happy with that idea. The care given to us here, they don't give that care to the adults. We want them to treat us equal. When sometimes I don't have money for transportation and am not able to come, they can even sack you but this place they don't do that."*<br>**Respondent 5 added:**<br>***"****I don't want to go to the adult clinic. I want to be here. I won't go anywhere"*<br>**Respondent 7 also shares:**<br>*"I'm ok with the adolescent, you see for the adult clinic on the other* hand, immediately *you enter there, people will be staring at you, like what's up with you, but the adolescent is not like that. Nobody even knows you are coming to the clinic."*<br>**Respondent 3 also added:**<br>*"The reason why I didn't want to leave the group is* because of the doctor who was *taking care of me, that was why I didn't want to leave the group to the adult, because I don't know the doctor, I'm going to meet to take care of me. That is why when he told me he was moving me to the adult clinic I didn't agree"*<br>**Respondent 4 also said that:**<br>*"I feel the work should improve "*

Experience with transiiton	***Attitude of Health Care workers***	Health care providers are a major stakeholder for the transitioning	*" The nurse sees you and starts straight away shouting and attacking you, they are like, " you don't like coming to clinic, you don't like taking your medicines but when you are sick then you come and sit here and worry us" It's not good. You don't know the problems of the person and the reasons that is preventing her from coming*<br><br>**Respondent 1 added:**<br>**"***They take care of us but like I said earlier their human relation with us is so poor"*<br>**Respondent 4 said:**<br>*"The nurses sometimes when you ask them questions, they don't mind you. Other times too when you ask them questions, they get angry."*
	Stigmatization		**Respondent 6 had this to say:**<br>*"I'm ok with the adolescent, you see for the adult clinic on the other hand, immediately you enter there, people will be staring at you, like what's up with you, but the adolescent is not like that. Nobody even knows you are coming to the clinic. Yea, maybe somebody you know is accompanying a patient and the person sees you. But with the adolescent, is mostly only the children and their mothers."*<br>**Respondent 8 added:**<br>*"I say bad because, I may get to the clinic late because I come from a far distance with bad roads inspite of this I get here early. I leave my job and come here early so that I can go back without anyone knowing I came here for check- up"*<br>**Respondent 7 added:**<br>*"The nurses should be patient and take their time when they are talking to the HIV patients when they come to clinic. During discussions they can talk to us calmly without them shouting for other people to hear. Because there are some people who have not disclosed their situations to any relative aside their parents and if such a person had come to the clinic with an aunty or a cousin using the mother as an excuse*

			that she's taking medicine for her, through the nurse's indiscretion, she will disclose her HIV situation to the relative and disgrace her"..
	Interference with School	their clinical visiting times and day falls on a weekday when they are supposed to be in class or at work	Respondent 6 commented:<br>*"It is somehow challenging because from the Friday to Monday sometimes you will be having class on Monday, but you have to skip the class and come on the Monday so as to see the doctor."*<br>**Respondent 5 also added**<br>*"I will just say the housing and the date set by the doctors because some of us are workers and students we must leave our class and work just to come here. If you are working with a private company the manager will not allow you. I will start my internship. I wonder how I will leave my internship course and come. Maybe my supervisor will be there, and I will wouldn't be around thereby dropping my GPA or something of that sort."*<br>**Respondent 8 also said:**<br>*"It is bad because, I may get to the clinic late because I come from a far distance with bad roads inspite of this I get here early. I leave my job and come here early so that I can go back without anyone knowing I came here for check-up, but I end up spending the whole day anytime I come"*
	Nepotism and Favouritism at the Clinic	Favouritism in which families and friends of health care providers are given preference and treated better	**Respondent 13 said:**<br>*"On the distribution of the drugs here. Sometimes, the place could be so full of people when you come, you know that some people came to meet you there, but they will serve the person before you. That is what I don't understand, they don't even ask for permission, for you to excuse them, probably he is going to do something, meanwhile you that you are sitting down too you are in a hurry to go and attend to something but they will be serving other people instead of you. So that is my big challenge that they should have a talk with them."*

		than the rest of the young adult	**Respondent 1 also added**:<br>*"Sometimes by the time you come a lot of people are here. Some will come late but bypass you to see the Doctor and that worries me a lot. We do form a queue. Sometimes it depends on the person's condition. Sometimes some of the clients know the Doctors and nurses so they just enter without joining the queue."*<br><br>*"I will say that both pharmacy and the nurses, when we the children come, they should focus on caring for us fairly rather than being bias and selective and jumping the queue for some people."*<br><br>**Respondent 3 said:**<br><br>*"There should be orderliness in the distribution of drugs. Some people come early because they want to leave early but those who come late will skip the queue and go for their drugs with the help of the staff whilst those who came early to form the queue will still be standing there"*
	Financial Challenges	Most of the ALHIV face financial challenges that sometimes causes them to default	Respondent 10 said:<br>*"When we are going to the laboratory, we need money to do it but sometimes the money too is not there, and my mother too will not be having. And the thing too it is the lab that will help you to know the level of the illness. So that is a big problem, sometimes I just decide not to come."*<br>It was seconded by Respondent13:<br>" *Sometimes when you have to come for review, he will tell you that there is no money, but what can you do, even if things are not good, he has to force and get money for you to come, because if you don't come and you default it will also be another problem*."

			R10, R5, R9 also added:<br>*"we don't always have money, sometimes feeding is a problem and we have to eat before we tale the drugs. That is why sometimes we default."*<br>According to respondent 3:<br>*"When I was at the childhood stage, they did something like when you come and you are leaving, they give you something like transportation fare, when your parents bring you and you are leaving, they them transportation fare".*
Improving Transition	***Sexual Reproductive Health Needs***	As ALHIV are maturing into adulthood there is the need to get into relationships and also engage in sexual activities.	Respondent 7 commented:<br>*"The little I will say is that our major problem as adolescents is the sexual reproductive health, STI's and pregnancy and those things. Some of us don't know what to do in the condition that they are in. For instance, you have a boyfriend that likes you, what should you do? Or she should abstain and if she can't, what can she do? There are people you will tell abstain, but if the person cannot abstain, the things that she can, you must tell the person."*<br>In addition to the above Respondent 13 added:<br>*"Ok, what can really help us is the adolescent sex and reproductive health. That is what I see as a big challenge for us the adolescents. Because at this stage, when you ask some people, they will not be able to answer. So, I will plead that some of the elders or some of the counsellors should be patient with us and make time and come and talk to us just as they get time to talk to the adults. Each and every day that we the children come so that it will also help us."*<br>Respondent 8, 4 and 1 also shared similar views.<br>*"As we become sexually active, they are a need for us to be taught how to protect ourselves from STI's and also avoid unwanted pregnancies since this can negatively influence treatments and spread"*<br>*Respondent 5, laments that:*

			"I have a boyfriend; we were together before I got to know why I was taking the medications. I have not told him anything, but we are having sex, am worried maybe he has it, but I don't even know what to do. No one is telling me anything and am afraid to ask since they will think a, a bad girl.
	Inadequate information on Treatment and Medication	ALHIV wanted to know more about the medications they take, how and when to take them, its benefits and what to do in case you are unable to take them at the stipulated time	Respondent 8 commented:<br>*"We need more information on the drug and the times to take the drug. In case you missed the time for taking the drug and whether you are to take the drug or to skip it. As for my drugs I take it before meals or after meals. So, the medications is also important."*<br>Respondent 3 had this say<br>*"They changed my medicine after they brought me to the adolescent clinic, the multiple pills taken at the paediatric clinic was reduced to a pill at the adolescent clinic, that is all I know. Its easier to take just the pill but mostly I forget".*<br>Respondent 7 had this to say:<br>*"I always make sure I take my drugs, what made me feel uncomfortable is that, if you have this disease you don't live for long in the world and also there are certain things you can't do it as the others, that is why I take my medication."*
	Privacy and Confidentiality	To reduce the fear of stigmatization that comes with disclosure, the health providers must ensure privacy for these young adults	Respondent 6 shared:<br>*"You know that adult clinic is for older people so moving from the young people to the old people, there are some who desire privacy before they share their thoughts but there are others who do not mind sharing their issues in the open so they has to be privacy in such instances. "*<br>Respondent 5 added:<br>*"There are discussions they can have with us calmly without shouting for the people to hear. Because there are some people who have not disclosed their situations to any relative aside their parents and if such a person had come to the clinic with an aunty*

			or a cousin using the mother as an excuse that she's taking medicine for her, through the nurse's indiscretion, she will disclose her HIV situation to the relative and disgrace her."<br>Respondent 9 said:<br>*"We need a lot of encouragement. There are some who think they are not healthy, but the staff have to tell them that the more you take your drugs the more you become healthy. That's better than taking the drugs sporadically without prompted of the dangers. Some are also unaware of the meaning of the lab tests (CB4, Viral load) that are undertaken and the purpose of medications: We need some more intense counselling"*<br>Respondent 2 also added:<br>*"They should teach and advise us more. When they advise us well, we wouldn't lack any knowledge concerning our health when we get to the adult clinic"*
	Creation of Support fund/ Foreign Aid	Some of the youth find it difficult to get transportation to the clinic, not to talk of money for their laboratory tests	Respondent 5 said:<br>*"Also, with regards to what you were saying you can get foreign aid to take them to school or abroad."*<br>Respondent 8 also added:<br>*"Also, it will be better for us if they put in more money. If they put in more money it will help to assist us a little so that when we are going to do the lab, it will not be a matter of whether you have or you don't have any money, and you don't have to struggle with going to do the lab".*<br>Respondent 5 also shared:<br>*"We need financial support".*<br>To the above, Respondent 7 also stated:<br>*"So I think what when you will do it will help us is when you find a particular time, and you gather all of us, either students or those who are into technical and vocational training and you ask us the work that we do, give them money or any necessary help*

			they need. For example, some are into vocational training and the madam ask her to even buy a simple comb, she doesn't have the money and you do that for her. Some are learning how to be hair dressers or even sew dresses and they've been asked to buy machines; they don't have that money unless the machine for the madam. But if the person is in adolescent and she tells them, they will do it for her. You are into vocational training and your parents don't have money; the adolescents will put you into the training free. Do you understand?"
	Provision of Experienced and Committed Health Care Providers	ALHIV complained of the poor treatment they receive from some health care providers especially the nurses.	Respondent 2 said: *"We need to have more Doctors"* Respondent 3 and 7shared: *"They should ensure that the drugs we are being given can weaken the HIV virus. If only they will care for us well and offer us our medication, it will help"* R1 and R 9 also said: *"The nurses sometimes when you ask them questions, they don't mind you. Other times too when you ask them questions, they get angry. I wish you could talk to them. The way they have been treating us in the adolescent's clinic, they should do the same thing for us in the adult clinic."* Respondent 7 also added: *"What you can add that will help is that the nurses, they should be patient and take their time when they are talking to the HIV patients when they come to clinic. There are discussions they can have with them calmly without them shouting for the people that they came with to hear*."
