# Supplementary material for: Adolescent on the bridge: Transitioning adolescents living with HIV to an adult clinic, in Ghana, to go or not to go?
Source: PLoS One. 2022 Sep 29;17(9):e0273999. doi: 10.1371/journal.pone.0273999 (PMC9522288; doi:10.1371/journal.pone.0273999)
Supplement: S1 Checklist — (DOCX) [file pone.0273999.s006.docx]

| **No. Item** | **Guide questions/description** |  | **Reported on Page #** |
| --- | --- | --- | --- |
| **Domain 1: Research team and reﬂexivity** |  |  |  |
| *Personal Characteristics* |  |  |  |
| 1. Inter viewer/facilitator | Which author/s conducted the interview or focus group? | RA | Methods -Page 4 |
| 2. Credentials | What were the researcher’s credentials? E.g. PhD, MD | RA - MPH, BSN, RGN  GM- PhD, MSc, BN  JN- PhD, MSc, BN | Methods Page 4 |
| 3. Occupation | What was their occupation at the time of the study? | RA- Registered Nurse, PhD Candidate  GM- Lecturer  JN- Lecturer | Page 4 |
| 4. Gender | Was the researcher male or female? | Females | N/A |
| 5. Experience and training | What experience or training did the researcher have? | At the time of the interviews, the researcher was a PhD student and had training in research and data collection as part of the program’s requirement. | N/A |
| *Relationship with participants* |  |  |  |
| 6. Relationship established | Was a relationship established prior to study commencement? | No | N/A |
| 7. Participant knowledge of the interviewer | What did the participants know about the researcher? e.g. personal goals, reasons for doing the research | Participants were provided with an information sheet which outlined the aim of the study. The participants and their guardians reviewed the participant information sheet prior to giving their written informed consent. | Methods – Page 4 |
| 8. Interviewer characteristics | What characteristics were reported about the interviewer/ facilitator? e.g. Bias, assumptions, reasons and interests in the research topic | Participants knew the researcher was a PhD candidate with an interest in the Transition process for ALHIV. | Additional File - S6 Information Sheet |
| **Domain 2: study design** |  |  |  |
| *Theoretical framework* |  |  |  |
| 9. Methodological orientation and Theory | What methodological orientation was stated to underpin the study? e.g. grounded theory, discourse analysis, ethnography, phenomenology, content analysis | Thematic content analysis | Methods- Page 4 |
| *Participant selection* |  |  |  |
| 10. Sampling | How were participants selected? e.g. purposive, convenience, consecutive, snowball | Purposive sampling | Methods- Page 3 |
| 11. Method of approach | How were participants approached? e.g. face-to-face, telephone, mail, email | Face-to-face Interviews were conducted | Methods- Page 4 |
| 12. Sample size | How many participants were in the study? | 13 | Results- Page 3 |
| 13. Non-participation | How many people refused to participate or dropped out? Reasons? | None | Results- Page 6 |
| *Setting* |  |  |  |
| 14. Setting of data collection | Where was the data collected? e.g. home, clinic, workplace | Komfo Anokye Teaching, Hospital | Methods- Page 3 |
| 15. Presence of non-participants | Was anyone else present besides the participants and researchers? | No | Methods- Page 4 |
| 16. Description of sample | What are the important characteristics of the sample? e.g. demographic data, date | - ALHIV between age of 13 to 19 years. - ALHIV on Outpatient care and enrolled in HIV care and taking ART. - Aware of HIV diagnosis - Already in care and transitioning or has been transitioned during the study period. | Results -Page 3 and 6 |
| *Data collection* |  |  |  |
| 17. Interview guide | Were questions, prompts, guides provided by the authors? Was it pilot tested? | Yes, participants were asked to reflect on their personal experience of the HIV program.  The questions were pilot tested amongst ALHIV in a different hospital. | Methods- Page 4 |
| 18. Repeat interviews | Were repeat inter views carried out? If yes, how many? | No | N/A |
| 19. Audio/visual recording | Did the research use audio or visual recording to collect the data? | Data were audio recorded using a digital recorder. | Methods-Page 4 |
| 20. Field notes | Were ﬁeld notes made during and/or after the interview or focus group? | No additional field notes were made | N/A |
| 21. Duration | What was the duration of the interviews or focus group? | Ranged between 60-90 minutes | Methods-Page 4 |
| 22. Data saturation | Was data saturation discussed? | Yes | Methods-Page 3 |
| 23. Transcripts returned | Were transcripts returned to participants for comment and/or correction? | Yes, feed-back and confirmation were sought from participants | Methods-Page 5 |
| **Domain 3: analysis and ﬁndings** |  |  |  |
| *Data analysis* |  |  |  |
| 24. Number of data coders | How many data coders coded the data? | 2 | Methods-Page 5 |
| 25. Description of the coding tree | Did authors provide a description of the coding tree? | Yes, supporting information (S2 Table) | Page 18 |
| 26. Derivation of themes | Were themes identiﬁed in advance or derived from the data? | Themes were derived from the data | Results- Page 8 |
| 27. Software | What software, if applicable, was used to manage the data? | Data were transcribed verbatim into Word documents by RA, and grouped by cutting and pasting between documents | Data Analysis Page 5 |
| 28. Participant checking | Did participants provide feedback on the ﬁndings? | No | Strengths and limitations Page 16 |
| *Reporting* |  |  |  |
| 29. Quotations presented | Were participant quotations presented to illustrate the themes/ﬁndings? Was each quotation identiﬁed? e.g. participant number | Yes | Results Page 8-13 |
| 30. Data and ﬁndings consistent | Was there consistency between the data presented and the ﬁndings? | Yes | Discussion Page 13 |
| 31. Clarity of major themes | Were major themes clearly presented in the ﬁndings? | Yes | Results 7-8 |
| 32. Clarity of minor themes | Is there a description of diverse cases or discussion of minor themes? | Yes | Discussion page 13 |

**Consolidated criteria for reporting qualitative studies (COREQ): 32-item checklist**

Developed from:

Tong A, Sainsbury P, Craig J. Consolidated criteria for reporting qualitative research (COREQ): a 32-item checklist for interviews and focus groups. *International Journal for Quality in Health Care*. 2007. Volume 19, Number 6: pp. 349 – 357
